# Imprime PGG-Mediated Anti-Cancer Immune Activation Requires Immune Complex Formation

**DOI:** 10.1371/journal.pone.0165909

**Published:** 2016-11-03

**Authors:** Anissa S. H. Chan, Adria Bykowski Jonas, Xiaohong Qiu, Nadine R. Ottoson, Richard M. Walsh, Keith B Gorden, Ben Harrison, Peter J. Maimonis, Steven M. Leonardo, Kathleen E. Ertelt, Michael E. Danielson, Kyle S. Michel, Mariana Nelson, Jeremy R. Graff, Myra L. Patchen, Nandita Bose

**Affiliations:** Biothera Pharmaceuticals Inc., Eagan, Minnesota, United States of America; Pusan National University, REPUBLIC OF KOREA

## Abstract

Imprime PGG (Imprime), an intravenously-administered, soluble β-glucan, has shown compelling efficacy in multiple phase 2 clinical trials with tumor targeting or anti-angiogenic antibodies. Mechanistically, Imprime acts as pathogen-associated molecular pattern (PAMP) directly activating innate immune effector cells, triggering a coordinated anti-cancer immune response. Herein, using whole blood from healthy human subjects, we show that Imprime-induced anti-cancer functionality is dependent on immune complex formation with naturally-occurring, anti-β glucan antibodies (ABA). The formation of Imprime-ABA complexes activates complement, primarily via the classical complement pathway, and is opsonized by iC3b. Immune complex binding depends upon Complement Receptor 3 and Fcg Receptor IIa, eliciting phenotypic activation of, and enhanced chemokine production by, neutrophils and monocytes, enabling these effector cells to kill antibody-opsonized tumor cells via the generation of reactive oxygen species and antibody-dependent cellular phagocytosis. Importantly, these innate immune cell changes were not evident in subjects with low ABA levels but could be rescued with exogenous ABA supplementation. Together, these data indicate that pre-existing ABA are essential for Imprime-mediated anti-cancer immune activation and suggest that pre-treatment ABA levels may provide a plausible patient selection biomarker to delineate patients most likely to benefit from Imprime-based therapy.

## Introduction

Imprime, a yeast derived soluble β-1,3/1,6 glucan (BTH1677); is currently in clinical development as an intravenously administered immunotherapy in combination with tumor-targeting, anti-angiogenic and immune checkpoint inhibitor antibodies. In a randomized phase II trial, first-line treatment of advanced non-squamous non-small cell lung cancer (NSCLC) patients with Imprime plus bevacizumab, carboplatin and paclitaxel yielded an overall response rate of 60.4% and a median overall survival of 16.1 months versus 43.5% and 11.6 months in patients treated with bevacizumab, carboplatin and paclitaxel (unpublished data). In high-risk chronic lymphocytic leukemia patients (including those with del 17p, del 11q risk factors), the addition of Imprime to rituximab and alemtuzumab yielded a complete response rate of 65% vs a 37% complete response in an earlier study at the same institution with only rituximab and alemtuzumab [1, 2].

In numerous preclinical tumor models, Imprime treatment enhanced the efficacy of tumor targeting and anti-angiogenic antibodies. In NSCLC xenografts, Imprime enhanced the anti-tumor efficacy of the anti-EGFR tumor-targeting antibody, cetuximab [3]. Imprime significantly enhanced the activity of an anti-MUC1 tumor-targeting antibody in a syngeneic T cell lymphoma model engineered to express the cell surface protein MUC1. Mechanistic work using this model revealed the critical role of complement protein C3, complement receptor 3 (CR3), and Gr1 positive myeloid immune cells in the anti-tumor activity of Imprime [4, 5]. In addition to enhancing the activity of tumor-targeting therapeutic antibodies, Imprime was also shown to enhance the anti-tumor efficacy of the anti-angiogenic antibody, bevacizumab, in ovarian and NSCLC cancer xenografts [6, 7].

**Fig 1:**
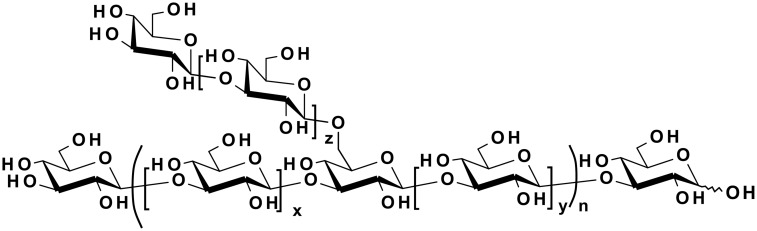
Structure of Imprime. Imprime is composed of a β-(1–3)-linked glucopyranose backbone with periodic β-(1–3)-linked glucopyranose side chains linked to the backbone via β-(1–6) glycosidic linkages.

Imprime is a soluble β glucan PAMP composed of a β-(1–3)-linked glucopyranose backbone with periodic β-(1–3)-linked glucopyranose side chains linked to the backbone via β-(1–6) glycosidic linkages (Fig 1). The geometrical nature of these β-(1–3)-linked glucose polymers allows the formation of triple helices held together via intermolecular hydrogen bonds and, in the case of Imprime, the triple helices further aggregate into higher order structures containing multiple triple helices. Imprime has been extensively purified, separated to a precise molecular weight, and well characterized [8].

In this study, we sought to explore the precise mechanism by which Imprime activates human innate immune cell functions to enhance the anti-cancer efficacy of monoclonal antibody therapy. Using whole blood (WB) from healthy human subjects, we now show that *ex vivo* Imprime treatment triggers a constellation of innate immune responses, including complement activation, chemokine production, and phenotypic activation of neutrophils and monocytes. Moreover, Imprime specifically elicits neutrophil-mediated reactive oxygen species (ROS) production and macrophage-mediated antibody-dependent cellular phagocytosis (ADCP). Imprime binding and functional activation of these innate effector cells requires that Imprime first form an immune complex with endogenous anti-β glucan antibodies (ABA). This complex then activates complement via the classical pathway, is subsequently opsonized by complement protein fragments and then engages both the CR3 and FcgRIIA receptors to elicit changes in innate effector functionality. These innate immune functions are only evident in the presence of sufficient ABA levels, which may suggest that ABA levels may represent a mechanism-based patient selection biomarker.

## Materials and Methods

### Antibodies and reagents

Antibodies and reagents used in the study are detailed in Table 1. Imprime PGG (BTH1677; β(1,6)-[poly-(1,3)-D-glucopyranosyl]-poly-β-(1,3)-D-glucopyranose; Biothera Pharmaceuticals Inc., Eagan, MN) was characterized as published previously [9]. Reagents used were as follows: Human AB serum (Gemini Bio-Products, West Sacramento, CA); purified human IgG and IgM (Athens Research & Technology, Athens, GA); C1q-depleted serum and C1q protein (Complement Technology, Inc., Tyler, TX); intravenous immunoglobulin (IVIG, Privigen^®^; CSL Behring, Kankakee, IL); glycine (Bio-Rad Laboratories, Inc., Hercules, CA); RPMI 1640 medium and Dulbecco’s PBS (dPBS) (Corning Cellgro, Manassas, VA); serum-free medium X-Vivo 10 (Lonza, Wakersville, MD); heat-inactivated Hyclone Characterized Fetal Bovine Serum (FBS), cytokine human 25-plex panel and Dynabeads untouched human monocytes kit (ThermoFisher Scientific, Pittsburgh, PA); TLR 7/8 agonist R848 (Invivogen, San Diego, CA); MicroVue CH50 Eq EIA kit, SC5b-9 Plus EIA kit, Complement C4a EIA kit and Complement C5a EIA kit (Quidel Corporation, San Diego, CA); Quantikine human IL-8 ELISA kit, recombinant human M-CSF and recombinant IL-10 (R&D Systems, Bio-techne, Minneapolis, MN); 6-plex and 14-plex Milliplex MAP kits (EMD Millipore, Billerica, MA); Compstatin (ICVVQDWGHHRCT) and control peptide (IAVVQDWGHHRAT) (Tocris Bioscience, Bristol, UK); EasySep Direct Human Neutrophil Isolation Kit (STEMCELL Technologies, Vancouver, BC, Canada); human cell lines HEK-293, Mantle cell lymphoma cell line Z138 and Burkitt’s lymphoma cell line Raji (ATCC, Manassas, VA). Whole glucan particles (WGPs) were derived and extensively characterized from a strain of *Saccharomyces cerevisiae* (Biothera Pharmaceuticals Inc.) [8]. All other chemicals were from Sigma-Aldrich Corp. (St. Louis, MO).

**Table 1:** List of antibodies used in the study.

Designation	Isotype	Specificity	Source
BfD IV (clone 10C6)	Murine IgM mAb	Yeast β-1,3/ 1,6-glucan	Biothera Pharmaceutical Inc.
FITC-conjugated F(ab’)_2_ Goat anti-Mouse IgM	Goat polyclonal	Mouse IgM	Southern Biotech (Birmingham, AL)
CD45 (clone HI30)	Murine IgG1 mAb	Human CD45	BioLegend (San Diego, CA)
CD14	Murine IgG1 mAb	Human CD14	BioLegend
CD15	Murine IgG1 mAb	Human CD15	BioLegend
Anti-C1q	Murine IgG1 mAb	Human C1q	Quidel
Anti-fD (clone 166–32)	Murine IgG1 mAb	Human factor D	ATCC
Anti-MBL (clone 3F8)	Murine IgG1 mAb	Human MBL	Dr. GL Stahl (Harvard Institute of Medicine, Boston, MA)
Anti-CD16 (clone 3G8)	Murine IgG1 mAb	Human CD16 (FcγRIII)	BioLegend
Anti-CD32 (clone AT-10)	Murine IgG1 mAb	Human CD32 (FcγRII)	AbD Serotec (Raleigh, NC)
Anti-CD32 (clone FUN-2)	Murine IgG2b mAb	Human CD32 (FcγRII)	BioLegend
Anti-CD64 (clone 10.1)	Murine IgG1 mAb	Human CD64 (FcγRI)	BioLegend
Anti-CD11b/CR3 (clone ICRF44)	Murine IgG1 mAb	Human CD11b/CR3	BioLegend
Anti-CD11a (clone LM2/1)	Murine IgG1 mAb	Human CD11a (I-domain)	Santa Cruz (Dallas, TX)
Anti-CD11a (clone VIM12)	Murine IgG1 mAb	Human CD11a (Lectin-like domain)	Santa Cruz
Anti-CD18 (clone IB4)	Murine IgG2a mAb	Human CD18	Ancell (Bayport, MN)
Anti-CR1 (clone 1B4)	Murine IgG1 mAb	Human CR1	Dr. Ronald P. Taylor (UVA School of Medicine, Charlottesville, VA)
Anti-C5b-9 (clone aE11)	Murine IgG2a mAb	Human C5b-9	Abcam (Cambridge, MA)
Anti-CD62L (clone DREG-56)	Murine IgG1 mAb	Human CD62L (L-selectin)	BioLegend
Anti-CD88 (clone S5/1)	Murine IgG2a mAb	Human CD88 (C5aR)	BioLegend
Anti-CXCR2 (clone 5E8/CXCR2)	Murine IgG1 mAb	Human CD182 (CXCR2)	BioLegend
Rituximab	chimeric Ab with human IgG1_κ_ constant domains	Human CD20	University of Minnesota-Boynton pharmacy
IgG1	Murine IgG1	Isotype control	eBioscience (San Diego, CA)
IgG2a	Murine IgG2a	Isotype control	eBioscience

### ELISA for ABA measurement

Human serum was prepared using Vacutainer^®^ SST^™^ tubes (Becton Dickinson, Franklin Lakes, NJ) according to the manufacturer’s instructions, and the cleared serum samples were used immediately or stored at –80°C. To measure relative concentration of serum IgG and IgM ABA in healthy subjects, IVIG supplemented sera or enriched ABA, a solid phase ELISA was developed in-house. Briefly, 1:2 serially diluted serum was incubated on a solid phase, 96-well microtiter plate pre-coated with Imprime. The plates were then incubated with either anti-human gamma chain or anti-human mu chain antibodies conjugated to peroxidase. A chromogenic substrate was subsequently added and the resulting color change was measured spectrophotometrically (absorbance at 450 nm). A commercially available pooled human serum was assigned an arbitrary value of relative antibody units per milliliter (RAU/mL) and run as a standard curve on each assay plate. Each healthy subject’s serum ABA concentration in RAU/mL was then correlated to the standard curve and corrected for the dilution factor.

### Immunoaffinity purification of ABA

IgG and IgM ABA were purified from pooled normal human plasma or human Ig using an Imprime-conjugated bead column as follows. To prepare Imprime—conjugated beads, sodium citrate buffer was exchanged with water from Imprime solution using a 10 kDa tangential flow Pellicon^®^ XL ultrafiltration cassette (EMD Millipore). Sodium periodate (G-Biosciences, St. Louis, MO) was added to a 10 mg/mL Imprime solution in water to achieve a level of 0.08 mg of oxidant per mg of Imprime. The mixed solution was placed on a rocker, protected from light, for 18 hrs to allow oxidation to occur. After incubation, the reaction mixture was diafiltered into water using an Amicon^®^ Ultra-15 centrifugal filter unit with an Ultracel-10 membrane (EMD Millipore). The oxidized Imprime was combined with washed UltraLink^®^ Hydrazide beads (ThermoFisher Scientific) to achieve a level of 10 mg beads per mg Imprime at a final concentration of 10 mg/mL oxidized Imprime in water. The resulting suspension was mixed for 18 hrs on a rocker at room temperature, washed 3 times with water by centrifugation, brought back to the same volume, and combined with sodium borohydride (J.T. Baker, Center Valley, PA) to achieve a level of 1 mg of sodium borohydride per 10 mg of beads. The resulting suspension was mixed and rocked as above for 18 hrs, washed 3 times with water by centrifugation, and packed into a 5-mL column (G-Biosciences). The control beads without Imprime were treated similarly. Human serum or human Ig were passed over the Imprime-conjugated or control bead column. The initial column volume was eluted and discarded, while the column flow-through containing ABA-depleted serum was collected and then quantified for the ABA levels by ELISA. IgG and IgM ABA retained on the Imprime-conjugated bead column were collected by washing the column twice with dPBS and then eluted with 0.1 M glycine•HCl (pH 3.3) (Bio-Rad Laboratories, Inc.). The eluate containing enriched ABA was neutralized with 0.1 mL of 1.0 M tris(hydroxymethyl)aminomethane (Tris; pH 8.5) for every mL of eluate, diafiltered and concentrated using an Amicon^®^ Ultra-15 centrifugal filter unit with Ultracel-10 membrane. The purified IgG and IgM were characterized by determining their concentration (RAU /mL) as described above. Purity was assessed by Western Blotting. Briefly, 2-ME-reduced protein samples were resolved by SDS-PAGE using a 4–20% Criterion Tris-HCL gel (Bio-Rad Laboratories, Inc.). The resolved gel was fixed, stained with Coomassie blue solution overnight, and then destained with destaining solution. For α-IgG and α-IgM immunoblot gel duplicates were prepared, resolved proteins were transferred onto nitrocellulose membranes (Bio-Rad Laboratories, Inc.) using a semi-dry transfer cell (Bio-Rad Laboratories, Inc.), the membranes were subsequently blocked with 5% non-fat dry milk prior to probing with HRP-conjugated goat anti-human IgG (gamma chain specific) or goat anti-human IgM (mu chain specific) Ab, respectively (Jackson ImmunoResearch Lab, West Grove, PA). The colorimetric detection was developed using the TMB membrane peroxidase substrate system (KPL Inc., Gaithersburg, MD).

### FcgR transfection of HEK-293

Plasmid DNA for human FcgRIIIB, FcgRIIA, and FcgRI (pCMV6-CD16b, pCMV6-CD32a, and pCMV6-CD64, respectively) was purchased from Origene (Rockville, MD). Transfection into HEK-293 cells was done using X-tremeGENE 9 DNA transfection reagent as per the manufacturer’s instruction (Roche, Mannheim, Germany), with the transfection rate of 80–100%. The cells were used for the study 48 hrs post transfection.

### Binding assays

Venous WB was obtained from healthy volunteers with informed consent at Memorial Blood Centers (St. Paul, MN) and at Biothera Pharmaceuticals Inc. as approved by NEIRB (New England Institutional Review Board), and incubated with Imprime at the described concentrations or with vehicle control at 37°C for the indicated time period. The bound Imprime was detected by flow cytometry using the glucan-specific mouse IgM mAb, BfD IV [10] and counterstained with FITC-conjugated F(ab')_2_ goat anti-mouse IgM secondary Ab. The gate for measuring the percentage of BfD IV positive cells for either monocytes or neutrophils was set using the vehicle treatment group to give < 2% of BfD IV positive cells and then applied to all treatment group(s) of that cell subset. For evaluation of binding in washed WB, washed WB with ABA addition, or WB with IVIG or ABA addition, binding was done with modifications. Washed WB was prepared by washing WB four times with large volume of dPBS, reconstituted with dPBS to the initial volume before evaluating Imprime binding. For ABA added to washed WB, indicated doses of ABA were added to washed WB prior to the correction to the initial volume with dPBS, and then followed by evaluating Imprime binding. Indicated amount of IVIG or ABA was added to WB and then evaluated for Imprime binding. Cross-over binding was done by adding the indicated volume of serum isolated from a subject with higher ABA levels to the recipient’s WB. To evaluate binding in the presence of blocking Abs, each blocking Ab (Table 1) was incubated with WB at 4°C for 30 mins prior to the incubation with Imprime at 37°C. Imprime binding in C1q-depleted serum with or without reconstitution with 100 ug/mL C1q recombinant protein was assessed in isolated neutrophils and monocytes as described previously [9]. For the HEK-293 transfectants, binding to the exogenous FcgR was assessed by incubating Imprime with 1 x 10^6^ cells as described. ABA on the Imprime-bound cell surface was detected with Alexa Fluor^®^ 647-AffiniPure F(ab’)_2_ fragment rabbit anti-human IgG Fc_γ_-specific Ab or Alexa Fluor^®^ 647-AffiniPure F(ab’)_2_ fragment rabbit anti-human IgM Fc_5μ_-specific Ab (Jackson ImmunoResearch Lab).

### Complement, cytokine and chemokine measurement

Serum CH50 measurement from healthy subjects and C4a, C5a and SC5b-9 production in the plasma of anti-coagulated WB (0.1 mL) treated with 10 μg/mL Imprime or vehicle, with or without IVIG addition for 30 mins at 37°C, were measured by ELISA using Microvue EIA kits according to the manufacturer’s instructions. Detection of CD11b, CD62L, CD88 and CXCR2 expression on neutrophils and monocytes after 10 μg/mL Imprime treatment of anti-coagulated WB for 30 mins at 37°C was determined using fluorescently-labeled antibodies listed in Table 1. Cytokine production was measured by Luminex (14-plex kit or 25-plex kit) in the plasma of anti-coagulated washed or unwashed WB (0.5 mL) treated with vehicle, 10 or 25 μg/mL Imprime and in the presence or absence of IVIG or ABA for 24 hrs. IL-8 and MCP-1 production were detected by the Luminex 6-plex kit or ELISA as indicated.

### Neutrophil-mediated reactive oxygen species (ROS) assay

ROS production was measured in a luminol-dependent chemiluminescence assay with modification as followed. Anti-coagulated WB was first treated with 25μg/mL Imprime or vehicle control in the presence or absence of indicated doses of ABA at 37°C for 2 hrs. Neutrophils were isolated via negative selection directly from WB and re-suspended in dPBS at between 2–3 x 10^6^ cells per well in a 96 well plate. These neutrophils were co-incubated with Raji B cell lymphoma cells (pretreated with or without 1μg/mL anti-CD20 Ab rituximab) at a 25:1 effector: target ratio. ROS generation was detected in the presence of luminol (50μM). Luminescence was read for 30 seconds intervals (30 secs to 90 mins).

### Macrophage-mediated antibody-dependent cellular phagocytosis (ADCP) assay

Monocyte-derived macrophages were prepared by *in vitro* as follows. WB was incubated with 25 μg/mL Imprime or vehicle control at 37°C in a 5% CO_2_ humidified incubator for 2 hrs. PBMC were isolated by ficoll separation followed by monocyte enrichment by negative selection using Dynabeads untouched human monocytes kit. Enriched monocytes were then cultured in XVivo10 media supplemented with 10% autologous serum with 50 ng/mL recombinant human M-CSF for 6 days. Recombinant human IL-10 was added prior to the last 72 hr of incubation. To measure macrophage-mediated ADCP, CellTrace violet-loaded Raji or Z138 lymphoma cells were incubated with rituximab (1μg/mL) at 4°C for 20 mins and then incubated with macrophages at 1:1 ratio at 37°C for 4 hrs. The cells were subsequently stained for CD11b and acquired by flow cytometry. ADCP was determined by calculating the percentage of cells positive for both CD11b and CellTrace violet (phagocytic macrophages) from the total number of CellTrace violet^+^ target cells.

### Determination of FcgRIIA polymorphism

Single nucleotide polymorphism (SNP) of FcgRIIA in healthy subjects was determined by PCR. Briefly, genomic DNA was isolated from 200 μL of WB using the QIAamp Blood Kit (Qiagen) according to the manufacturer’s instruction. 100 ng of genomic DNA was used in PCR reactions specific for HIS or ARG FCGR2A alleles using the QuantiTect SYBR Green PCR Kit (Qiagen). Primer sequences of H131-specific and R131-specific forward primers were 5’-AAAATCCCAGAAATTCTCCCA-3’ and 5’-AAAATCCCAGAAATTCTCCCG-3’, and the reverse primer is 5’-TCCTGTGCAGTGGTAATCAC-3’.

### Statistical analysis

Prism software version 6 (GraphPad Software, Inc., La Jolla, CA) was used for all statistical analysis. Statistical comparisons of treatment groups were performed with the Student’s t-test and correlation (r) between data sets were determined by Pearson correlation efficient. *P* <0.05 was considered statistically significant. *P < .05; ** P < .01; *** P < .005; **** P < .0001.

## Results

### Classical complement pathway activation is required for Imprime binding

**Fig 2:**
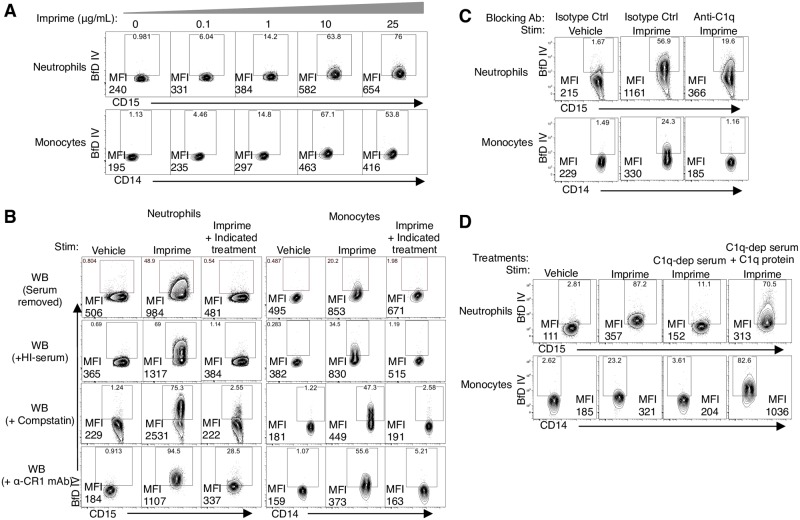
Imprime binding to neutrophils and monocytes in whole blood is complement-dependent. (A) Binding of increasing concentration of Imprime (0, 0.1, 1, 10, and 25 μg/mL) to human neutrophils and monocytes was measured by flow cytometry after incubation of WB with Imprime or vehicle at 37°C for 30 mins. (B) Imprime binding in the absence of serum (serum removed), heat-inactivated serum (HI serum), presence of anti-C3 peptide, compstatin (100 μM) or α-CR1 mAb (10 μg/mL) (right column in each respective row) are compared to the binding to vehicle or Imprime of untreated WB (left and middle column in each respective row). For compstatin and α-CR1 mAb treatment, WB was pre-treated with the blocking agents at 4°C for 30 mins prior to the incubation with 10 μg/mL Imprime at 37°C for 30 mins. (C) The role of classical pathway in Imprime binding to neutrophils and monocytes was evaluated by blocking with the anti-C1q mAb (50 μg/mL) at 4°C for 30 mins prior to the incubation with Imprime or vehicle. (D) Imprime binding to enriched human neutrophils and monocytes in 20% serum, 20% C1q-depleted serum (C1q-dep serum), or 20% C1q-depleted serum replenished with 100 μg/mL purified human C1q protein (C1q-dep serum + C1q protein) was determined by flow cytometry. The MFI and percentage of BfD IV positive cells are indicated on the contour plots. Data shown for each part are representative of 3–5 independent experiments performed with different donors.

We have previously shown that Imprime binding to isolated neutrophils and monocytes requires complement [9]. In this study, we sought to characterize Imprime binding to these cell subsets in human WB, a more physiologically-relevant experimental condition. Binding increased with increasing concentrations of Imprime at 0.1, 1, 10, and 25 μg/mL (Fig 2A). Imprime binding was completely inhibited in the absence of serum and when serum was heat inactivated, supporting the necessity of complement. Imprime binding to both neutrophils and monocytes was inhibited by blocking complement activation using either Compstatin, a C3-specific inhibitor or anti-complement receptor 1 (CR1), the cofactor required for conversion of complement fragment C3b to iC3b (Fig 2B). It is also important to note that the baseline level of Imprime binding is variable in different donors.

We next sought to delineate the role of different complement activation pathways in Imprime binding using blocking mAbs against C1q, the mannose-binding lectin (MBL) protein and Factor D to specifically block the classical, lectin and alternative pathways, respectively. Imprime binding to both neutrophils and monocytes was substantially inhibited by the anti-C1q antibody (Fig 2C). Binding of Imprime was significantly reduced when in C1q-depleted serum but was restored by C1q supplementation (Fig 2D). In contrast, the anti-factor D antibody only partially inhibited binding, while the anti-MBL antibody had no effect. Imprime binding (S1 Fig). These data collectively demonstrated that classical complement pathway is critical for Imprime binding to innate immune cells.

### Imprime forms an immune complex with ABA in human serum

**Fig 3:**
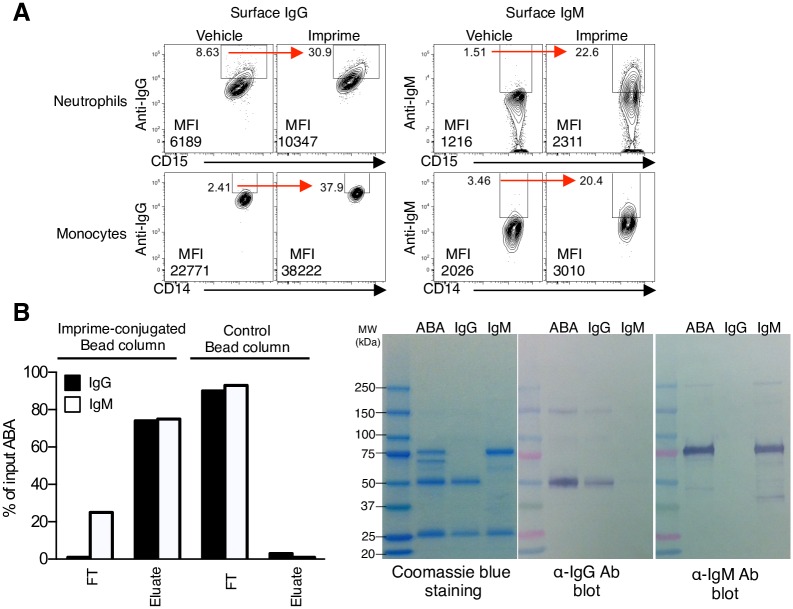
Imprime interacts with endogenous IgG and IgM ABA in human serum. (A) The surface IgG and IgM ABA on vehicle- or Imprime-treated neutrophils and monocytes were detected by flow cytometry using rabbit anti-human IgG- and IgM-specific Abs, respectively, after Imprime was incubated with WB at 37°C for 30 mins. The MFI and percentage of IgG- or IgM-positive cells are indicated on the contour plots. Data shown are representative of 3 independent experiments. (B) The ability of an Imprime-conjugated bead column to selectively bind and retain IgG (filled bar) and IgM (empty bar) ABA from human serum is shown. The percentage of ABA in the flow-through (FT) and the eluate was measured based on the serum ABA concentration pre-loaded on the column. The ABA eluted off the Imprime-conjugated bead column along with purified IgG and IgM controls were resolved by SDS-PAGE under reducing conditions. Protein bands corresponding to the heavy and light chain of IgG and IgM were observed by Coomassie staining (left panel), or detected by immunoblotting with an anti-IgG Ab (middle panel) or anti-IgM heavy chain Ab (right panel).

The classical pathway for complement activation is primarily initiated by antigen-IgG/IgM antibody immune complexes. We therefore hypothesized that Imprime must be opsonized by ABA, and as such, ABA would be detectable on the cell surface of Imprime-bound cells. By flow cytometry, we show increased cell surface staining for IgG, IgM or both from healthy subjects only after incubation with Imprime (Fig 3A) suggesting formation of these Imprime-ABA complexes.

The ability of serum ABA to complex specifically with Imprime was subsequently evaluated. Pooled human serum was passed over an Imprime-conjugated bead or a control bead column. The concentrations of the IgG and IgM ABA in the flow through (FT) and the eluate were measured relative to the total ABA in the pre-loaded serum (% input). The eluate from the Imprime-conjugated bead column contained approximately 75% of the ABA present in the serum. Only minimal ABA were evident in the eluate of the control column. This indicated that the ABA was specifically retained on the Imprime-conjugated column. The enriched ABA from the eluate of the Imprime-conjugated bead column was characterized by SDS-PAGE and identified by Western blotting. Corresponding to the bands seen with the purified IgG and IgM controls, the heavy and light chains of IgG (50 kDa and 25 kDa, respectively) and IgM (75 kDa and 25 kDa, respectively) ABA were observed (Fig 3B). Taken together, these data support the notion that Imprime forms immune complexes with both IgG and IgM ABA.

### Imprime binding requires ABA

**Fig 4:**
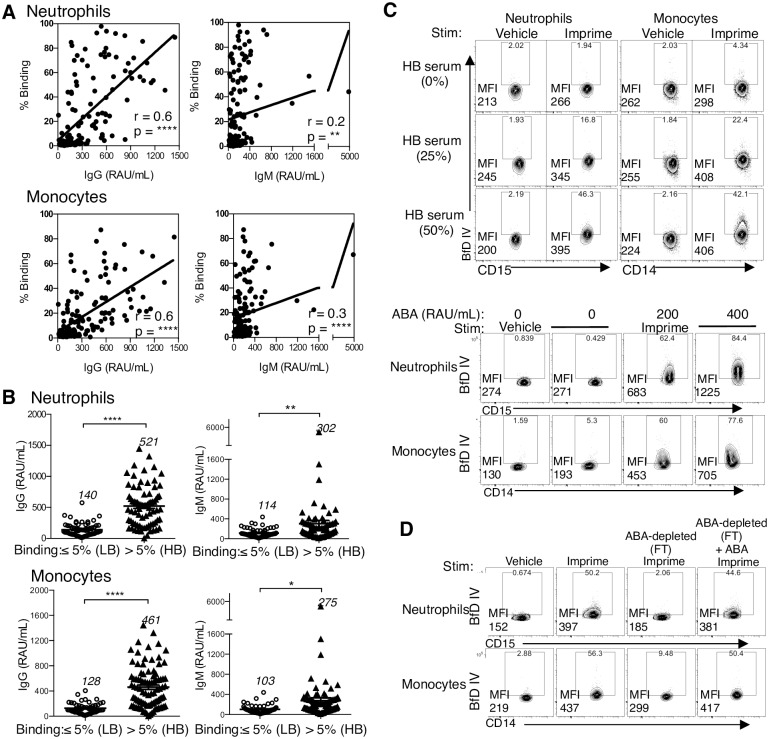
Imprime binding requires ABA. (A) Imprime binding (10 μg/mL) was measured in WB from 143 healthy donors. The serum from the same blood donation was used to measure relative ABA concentrations by ELISA with RAU/mL as the reportable value. The Pearson’s correlation (r) and *P*-value of Imprime binding to neutrophils and monocytes binding and ABA IgG and IgM concentrations are shown. (B) The IgG and IgM ABA level of each of the 143 healthy subjects identified as HB (>5% binding) and LB (≤ 5% binding) are shown in the scatter plot with the mean IgG or IgM ABA concentration of each group indicated. Imprime binding in a LB was evaluated by (C) adding increasing amount of HB serum and increasing concentration of ABA. (D) Imprime binding in a HB was evaluated in ABA-depleted serum (FT; flow through from the Imprime-conjugated bead column) and subsequently rescued binding by exogenous supplementation of ABA. MFI and percentages of BfD IV-positive cells are indicated on the contour plots. Data presented in (C) and (D) are representative of at least 3 independent experiments performed with different donors.

In investigating Imprime binding in WB from healthy subjects, we observed substantial variability in binding between different subjects. Since ABA levels vary across the human population [11–13], we sought to characterize whether differences in ABA levels may influence Imprime binding. In sera from 143 healthy human volunteer subjects, binding of Imprime in WB was correlated to IgG (independent of IgM) or IgM (independent of IgG) ABA levels measured in the serum from the same blood draw. The Pearson’s correlation between IgG ABA concentration and neutrophil or monocyte binding (r = 0.6 for both neutrophil and monocyte binding) was stronger than that observed for IgM ABA levels (r = 0.2 for neutrophil binding and 0.3 for monocyte binding) (Fig 4A). For further insight into binding differences, we attempted to segregate the subjects into ‘low binder(s)’ and ‘high binder(s)’ based on the extent of Imprime binding. For low binders (LB), Imprime consistently bound less than 5% of the total neutrophils or monoctytes. In high binders (HB), Imprime bound to 5–97.9% neutrophils, while monocyte binding ranged from 5–87.3%. Of the 143 healthy subjects, approximately 65% were identified as Imprime HB (~80% of the HB were high for both neutrophil and monocyte binding). The distribution curves and frequency tables are presented in S2 Fig and S1 and S2 Tables. As shown in Fig 4B, the mean ABA IgG and IgM concentrations were significantly higher in HB.

We then investigated whether the level of ABA may be critical for Imprime binding and may be the key differentiating factor between HB and LB. We first ruled out the possibility that the HB and LB groups show differential Imprime binding due to inherent differences in classical pathway complement activation using the CH50 test, the screening test for total complement activity. There were no differences in CH50 across groups suggesting both were similarly capable of classical complement pathway activation (S3A Fig). We next evaluated whether LB show lower cell surface Imprime binding due to greater internalization of surface bound Imprime. LB showed only minimal Imprime binding at both 4°C and 37°C suggesting that quicker internalization was not responsible for lower cell surface binding in LB (S3B Fig). We then hypothesized that LB may have insufficient ABA levels to enable complex formation with Imprime. As shown in Fig 4B, the mean ABA IgG and IgM concentrations were significantly lower in LB than in HB. Supplementing a LB’s whole blood cells with HB sera (i.e. sera with higher ABA) resulted in a dose-dependent increase in Imprime binding as did supplementation with enriched ABA (Fig 4C). Moreover, depletion of ABA from HB whole blood reduced Imprime binding but could be rescued by add-back with enriched ABA (Fig 4D). Together, these data confirm that sufficient ABA levels are crucial for complex formation with Imprime and subsequent binding to the innate immune cells.

### FcgR, in addition to CR3, plays a role in binding of the Imprime-ABA immune complex

**Fig 5:**
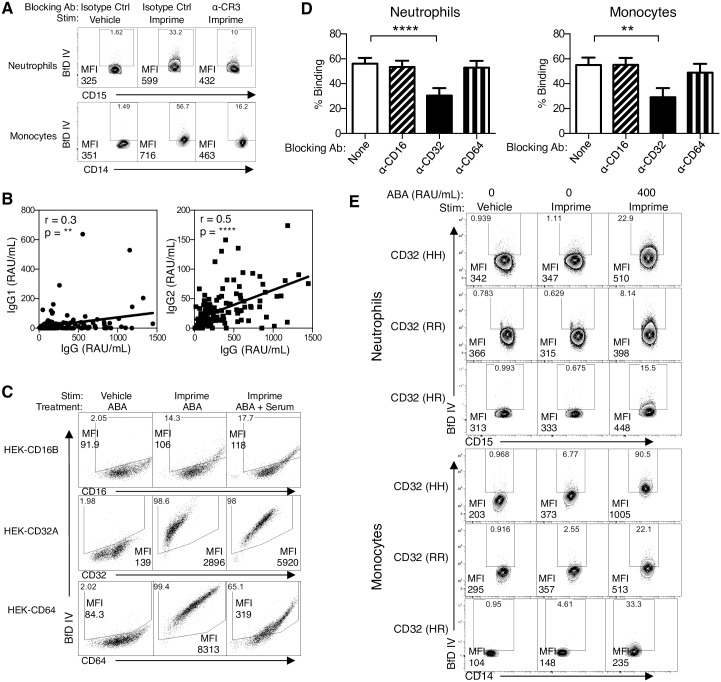
Role of CR3 and FcgRIIA in binding of Imprime. (A) Imprime binding was measured in the presence of a combination of α-CR3 blocking mAbs (LM2/1, a mAb against the I-domain of CR3 at 10 μg/mL; VIM12, a mAb against the lectin-domain of CR3 at 10 μg/mL; IB4, a mAb against the β2 chain of CR3 at 5 μg/mL). WB was incubated with the antibodies at 4°C for 30 mins prior to the incubation with 10 μg/mL Imprime at 37°C for 30 mins. Representative results from 3 independent experiments are shown. (B) Total serum IgG ABA level from 143 healthy subjects was measured by ELISA as described in Fig 4. Subclass ELISA was performed similarly with the exception of using IgG1 and IgG2 specific secondary antibodies. The Pearson’s correlation (r) and *P*-value of total IgG ABA and IgG1 or IgG2 ABA are shown. (C) Plasmid DNA for individual FcgR, including FcgRIIIB (CD16B), FcgRIIA (CD32A), and FcgRI (CD64) as well as vector DNA (data not shown) was transfected into HEK293 cells, and Imprime binding was then assessed in these FcgR—expressing HEK293 transfectants by incubating with 10 μg/mL Imprime and enriched ABA (100 RAU/mL) in the presence or absence of serum at 37°C for 30 mins. Representative results from 3 independent experiments are shown. (D) Imprime binding in WB was assessed in the presence of respective FcR blocking mAbs, 20 μg/mL of anti-CD16 (FcgRIII), anti-CD32 (FcgRII), or anti-CD64 (FcgRI) by flow cytometry. Data are shown as mean ± SEM of multiple donors (N = 12). (E) Imprime binding in the presence of enriched ABA (400 RAU/mL) was measured in 3 LB identified to have H131 (HH) or R131 (RR) or the heterozygous HR allotype of FcgRIIA by PCR. Representative results are shown here from 3 independent experiments.

We have previously shown that when the known receptors for β-glucan, including CR3 and Dectin-1were blocked, anti-CR3 antibodies were the most effective at inhibiting Imprime binding to isolated neutrophils and monocytes [9]. In this study, we confirmed the role of CR3 in Imprime binding to the same cell subsets in whole blood as antibodies blocking the CD11b and the CD18 chains of CR3 inhibited binding to both neutrophils and monocytes (Fig 5A).

With the new understanding that Imprime binding also requires complex formation, particularly with IgG ABA, we hypothesized that the FcgR may also play a role in Imprime binding. Given that the human IgG responses to carbohydrate antigens are primarily restricted to the IgG2 subclass, and that FcgR have varying affinities for different subclasses of IgG [14–16], we first evaluated the IgG ABA subclass that is most reactive to Imprime. Relative levels of IgG1 and IgG2 in serum from 143 healthy subjects were measured and correlated with the corresponding total IgG ABA levels. A correlation of r = 0.5 was observed between the IgG2 level and total ABA concentration while the correlation between the IgG1 ABA level and total ABA concentration was weaker (r = 0.3) (Fig 5B) suggesting that the majority of IgG ABA was IgG2.

We next evaluated the role of FcgR in binding of the Imprime-ABA immune complex. Human neutrophils predominantly express CD32A (FcgRIIA) and CD16B (FcgRIIIB), while monocytes express CD32A and CD64 (FcgRI) [15, 16]. We hypothesized that CD32A, the sole FcgR capable of binding multimeric IgG2 with higher affinity [17, 18], would play a critical role in binding of Imprime-ABA complex binding. We transiently transfected HEK293 cells to overexpress each of these FcgR. In the absence of serum, Imprime-ABA immune complexes formed by exogenously adding enriched ABA bound to FcgRIIA and FcgRI, In the presence of serum (i.e., physiological condition) binding to FcgRI drastically diminished but binding to FcgRIIA was sustained. Imprime-ABA showed minimal binding ability to the other low affinity receptor, FcgRIIIB, regardless of the presence or absence of serum (Fig 5C).

The role of FcgR in binding of Imprime in whole blood was next evaluated for each FcgR using specific blocking antibodies. Consistent with the observations using the HEK293 overexpression system, Imprime binding to neutrophils and monocytes in WB was significantly inhibited only by the FcgRII blocking antibody (Fig 5D). We next sought to determine whether the individuals with the known genetically determined allotypes of FcgRIIA—the products of H131 and R131 alleles- would show differential binding to Imprime-IgG2 immune complexes. Both neutrophils and monocytes in individuals with FcgRIIA-H131 have shown to be effective in interaction with IgG2, whereas those with FcgRIIA-R131 show weaker binding of IgG2 [17, 19]. Imprime binding was measured after addition of enriched ABA to WB of LB identified as H131 (HH), R131 (RR) or heterozygous (HR) allotype by the PCR assay. As expected, the addition of equal amounts of ABA resulted in significantly higher binding of Imprime to both neutrophils and monocytes in subjects with the H131 (HH) versus the R131 (RR) allotype; the heterozygous (HR) allotype showed intermediate binding (Fig 5E).

### Imprime activates innate immune functions

**Fig 6:**
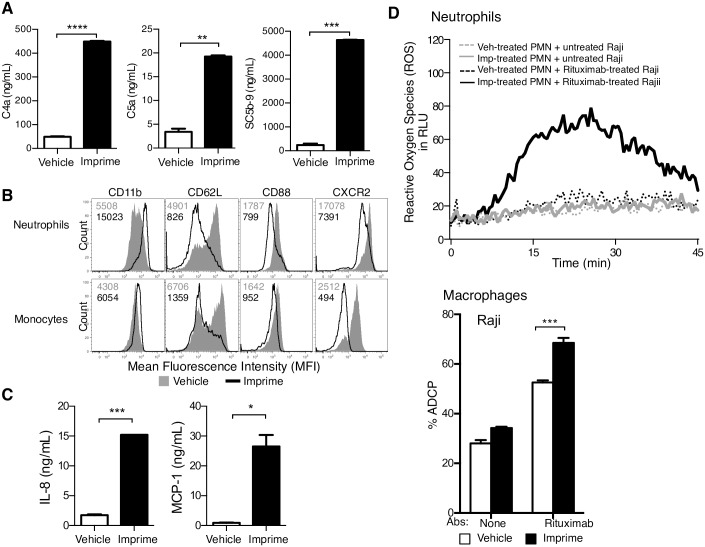
Imprime activates innate immune functions. (A) Complement activation proteins C4a, C5a, SC5b-9 in the plasma of WB treated with 10 μg/mL Imprime or vehicle for 30 mins at 37°C was measured by ELISA. Data represent mean ± SEM of triplicates for each treatment condition. (B) Modulation of CD11b, CD62L, CD88 and CXCR2 expression on neutrophils and monocytes post Imprime binding in WB was determined by flow cytometry. (C) Chemokine, IL-8 and MCP-1, production in the plasma of WB treated with Imprime or vehicle for 24 h at 37°C was measured by Luminex. Data represent mean ± SEM of duplicates from 3 independent experiments. (D) ROS production in 25:1 co-cultures of neutrophils (isolated from WB treated with 25 μg/mL Imprime or vehicle for 2 hrs at 37°C) and Raji cells treated with or without 1 μg/mL rituximab was measured by luminescence-based assay. Macrophage-mediated ADCP was measured by flow cytometry after 1:1 co-incubation of macrophages (differentiated from monocytes isolated from WB treated with 25 μg/mL Imprime or vehicle for 2 hrs at 37°C) with Raji cells treated with or without 1 μg/mL rituximab. Representative results are shown here from at least 3 independent experiments performed with three different donors.

We next sought to characterize the ability of Imprime to activate known early innate immune responses typically triggered by PAMP recognition- complement activation, phenotypic activation of neutrophils and monocytes, and cytokine production. The complement activation proteins C4a, C5a, and SC5b-9 were significantly increased in the plasma of HB healthy subjects 30 mins after *ex vivo* Imprime treatment (Fig 6A). As fluid phase terminal complement complex, SC5b-9 was detected in the plasma, we checked for the membrane-bound C5b-9, but it was not detected on the surface of either neutrophils or monocytes (S4 Fig). Several phenotypic changes associated with monocyte and neutrophil activation were measured 30 mins after Imprime treatment of WB. Both neutrophils and monocytes showed increased expression of CD11b concomitant with decreased expression of CD62L (L-selectin), CD88 (C5a Receptor), and CXCR2, a receptor involved in transendothelial migration (Fig 6B). Cytokine evaluation showed that Imprime, in contrast with the positive control TLR 7/8 agonist, only consistently induced the specific chemokines, IL-8 and MCP-1 (Fig 6C and S3 Table).

These data demonstrate that Imprime treatment can activate innate immune cell function. We therefore chose to evaluate whether Imprime may also activate the anti-cancer activity of these innate immune effectors by assessing neutrophil-mediated generation of ROS as well as macrophage- mediated ADCP. When incubated with rituximab-opsonized Raji cells, neutrophils isolated from HB whole blood after 30 min of Imprime treatment showed a remarkable increase in the generation of ROS. Likewise, when co-cultured with rituximab-bound Raji cells, macrophages differentiated from WB-isolated monocytes showed increased tumor cell phagocytosis. In the absence of rituximab, Imprime-treated neutrophils and macrophages showed minimal ROS generation and ADCP, respectively (Fig 6D). Tumor cells without antibody (rituximab) failed to elicit substantive neutrophil-mediated ROS generation or macrophage-mediated ADCP (Fig 6D) suggesting that Imprime-activated tumor cell killing requires antibody opsonization of the tumor cell.

### Imprime-mediated innate immune effector functions require ABA

**Fig 7:**
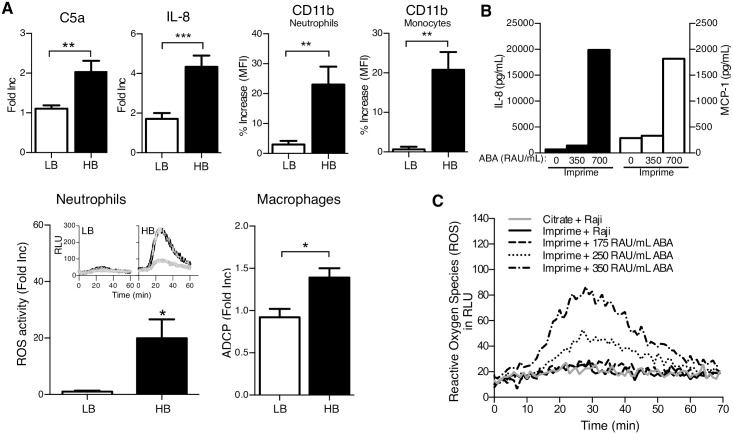
ABA are critical for Imprime function. (A) The generation of C5a in WB plasma (14 LB and 18 HB) after incubation with Imprime for 30 mins, and IL-8 (21 LB and 11 HB) in WB after incubation with Imprime for 24 hrs were measured by ELISA. The amounts of C5a or IL-8 produced by Imprime—treated WB are presented as fold increase for individual HB and LB relative to the vehicle control. CD11b expression on neutrophils (11 LB and 12 HB) and monocytes (8 LB and 10 HB) after incubation with Imprime for 30 mins were assessed by flow cytometry. The % increase of CD11b was calculated using the MFI of Imprime-treated cells compared with vehicle-treated cells as baseline. ROS and ADCP assays were performed as described in Fig 6. The amount of ROS activity is presented as fold increase of AUC relative to that of the vehicle-treated neutrophils. The insets show the representative ROS activity of neutrophils from a LB or a HB over a 60-mins time period. The ADCP is presented as average fold increase relative to the vehicle control. Results for ADCP and ROS are from 3 HB and 3 LB. Data represent mean ± SEM of triplicates for each treatment condition. IL-8 and MCP-1 production (B) and neutrophil ROS activity (C) were assessed in LB after exogenous addition of ABA to Imprime-treated WB. Results presented are representative of at least 3 independent experiments performed with different donors.

As there was a significant correlation between Imprime binding and higher ABA levels in HB vs LB, we next evaluated whether Imprime-induced innate immune activation was also different between the HB and LB. As shown in Fig 7A, C5a and IL-8 production in plasma, as well as increased CD11b on neutrophils and monocytes, were significantly higher in HB. Additionally, only in HB did we see substantially increased neutrophil-mediated ROS production and macrophage-mediated ADCP.

As binding in LB could be rescued by supplementation with HB serum or by exogenous addition of enriched ABA, we investigated whether Imprime-activated functions in LB could also be rescued in a similar manner. Fig 7B shows that Imprime was able to induce chemokine production in LB only upon addition of exogenous ABA. Likewise, Imprime was also able to induce increased neutrophil-mediated ROS production in LB in an ABA concentration-dependent manner (Fig 7C).

**Fig 8:**
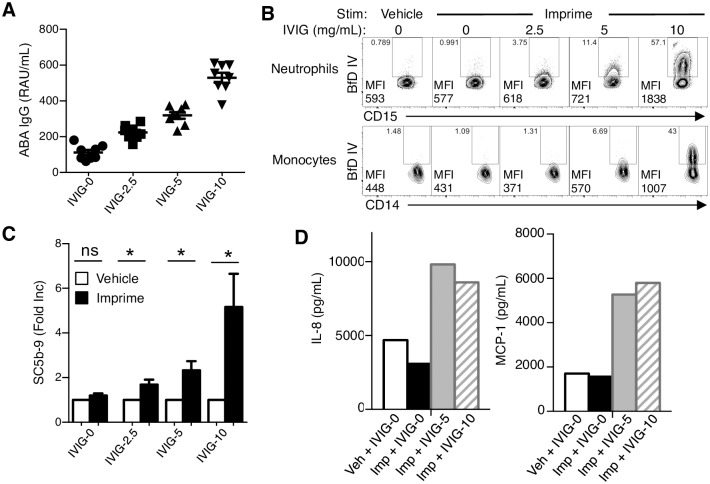
IVIG addition rescues Imprime binding and function in LB by increasing ABA levels. (A) Plasma isolated from WB samples spiked with PBS (IVIG-0) or IVIG at final concentrations of 2.5 mg/mL (IVIG-2.5), 5 mg/mL (IVIG-5) and 10 mg/mL (IVIG-10) was analyzed for IgG ABA antibodies by ELISA. Data represent mean ± SEM of 8 subjects. WB from LB were treated with vehicle control or 10 μg/mL Imprime in the presence of the indicated IVIG concentrations and then assessed for, (B) Imprime binding to neutrophils and monocytes by flow cytometry, (C) induction of SC5b-9 by ELISA after 30 mins and (D) chemokine production by Luminex after 24 hrs. Results are representative of 3 independent experiments performed with different donors.

The ability of ABA supplementation to restore Imprime binding and innate immune functions in LB led us to consider the potential clinical ramifications of this finding. We investigated whether intravenous formulation of immunoglobulin (IVIG) could also rescue LB functional response to Imprime. IVIG preparations are sterile, purified IgG products manufactured from the pooled plasma of thousands of blood donors and commonly used in a variety of clinical settings to support immune function [20]. The level of ABA in any pooled preparation of IgG would represent the mean value of the donor population. As shown in Fig 8A, addition of IVIG to the whole blood of LB increased ABA levels in a dose-dependent manner. Increased ABA levels by the spiked IVIG enabled complement activation, Imprime binding to both neutrophils and monocytes, and the production of chemokines in the LB (Fig 8B–8D). Collectively, these data suggest that ABA are critical for the ability of Imprime to activate innate effector function and that a threshold ABA level, which could be reached by IVIG or purified ABA supplementation in those with naturally low ABA, may be necessary for Imprime to act therapeutically.

### Both CR3 and FcgRIIA are critical for the complete Imprime-mediated innate activation

**Fig 9:**
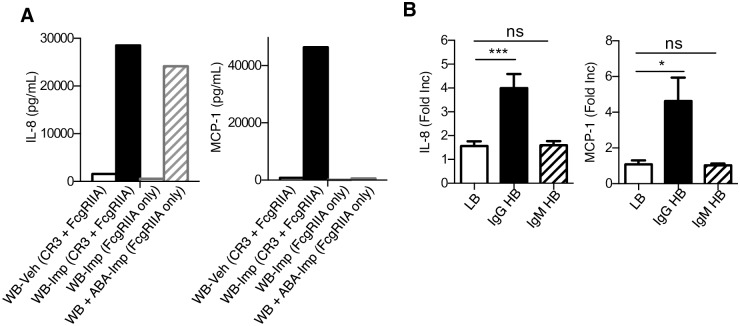
CR3 and FcgRIIA play a critical role in Imprime’s functional response. (A) IL-8 and MCP-1 production in the culture supernatant of complement-intact (CR3 + FcgRIIA) and complement-free/ABA supplemented (FcgRIIA only) WB of a HB were measured 24 hrs after Imprime binding. Results are representative of 3 independent experiments performed with three different donors. (B) IL-8 and MCP-1 production in 64 LB, 59 IgG HB (higher IgG ABA) and 6 IgM HB (higher IgM and lower IgG ABA) were measured after 24 hrs by ELISA and luminex. Data represent mean ± SEM of the number of binders indicated above.

In order to dissect the role of FcgRIIA and CR3 in Imprime-mediated functions, we investigated whether there were differences in functional activities induced by Imprime-ABA immune complexes upon engagement of FcgRIIA alone (complement-free) versus both CR3 and FcgRIIA (complement-intact). Binding of Imprime-ABA via FcgR alone was tested in a complement-free setting by washing away the plasma from the WB, and replacing with an equivalent volume of dPBS containing only enriched ABA. In this setting, anti-FcgRII blocking antibody completely inhibited binding of Imprime-ABA immune complex. No inhibition was observed with the anti-CR1 antibody, indicating that binding was only FcgRIIA- and not CR1-mediated (S5A Fig). We then compared Imprime’s ability to induce chemokines in this complement-free (FcgRIIA alone) setting versus complement-intact whole blood, where both CR3 and FcgRIIA engagement would be expected. IL-8 was produced in both complement-intact and complement-free WB, indicating that Imprime binding to FcgRIIA alone was sufficient to stimulate IL-8 production (Fig 9A). However, MCP-1 was induced only in the presence of complement, indicating a requirement for both CR3 and FcgRIIA. We further investigated whether Imprime binding of CR3 alone can drive similar functionality. As it was difficult to experimentally simulate this condition in whole blood, we identified 6 subjects out of the 143 subjects with higher IgM but lower IgG ABA levels. For these subjects, Imprime would be complexed predominantly with IgM ABA. As such binding of these complexes would not be mediated by FcgRIIA engagement but rather would reflect only CR3–mediated binding (S5B Fig). As expected, WB from these subjects did not show any chemokine induction (Fig 9B). These data collectively show that both CR3 and FcgR are necessary for full Imprime-triggered innate immune functionality.

## Discussion

Imprime has been well tolerated when administered intravenously to more than 300 cancer patients and has consistently shown compelling improvements in clinical response in multiple phase 2 clinical trials in combination with tumor-targeting or anti-angiogenic antibody therapies [1, 21]. In numerous preclinical tumor models, Imprime has repeatedly demonstrated anti-tumor efficacy in combination with tumor targeting, anti-angiogenic and immune checkpoint inhibitor antibodies [3–7]. Mechanistically, Imprime acts as a PAMP to trigger activation of innate immune effector cells, which then orchestrate a coordinated anti-cancer immune response with cells of the adaptive immune system. Understanding the precise mechanisms by which Imprime binds to innate effector cells, eliciting phenotypic and functional activation is essential to realizing the therapeutic utility of Imprime.

In this study, we now show that Imprime binding and functional activation require first the formation of an immune complex with naturally occurring ABA. Imprime, a 1,3/1,6- β glucan isolated from the cell wall of *Saccharomyces cerevisiae* is a natural product, and is readily recognized by the innate immune system as a “non-self” danger signal, or PAMP. It is therefore not surprising that naturally occurring antibodies to such a β glucan would exist, as these antibodies may reflect a hummoral immune response to prior fungal infections. Varying levels of different isotypes of ABA, including IgG, IgM, and IgE, have been measured in human serum in different studies [12, 13]. Our data show that Imprime complexes mainly with IgG and IgM isotypes of ABA; it is also possible that Imprime complexes with other ABA isotypes, but detailed investigations of these interactions were beyond the scope of this study.

Typically, antibodies to biologic therapies are thought to neutralize the efficacy of the biologic therapy [22]. Remarkably, for the therapeutic action of Imprime, these naturally occurring ABA are essential, as Imprime-mediated activation of innate immune effector cells is dependent upon the formation of an ABA-Imprime immune complex. Imprime binding and innate immune activation is most evident in subjects with higher pre-existing levels of ABA (HB), particularly ABA of the IgG isotype. Depletion of ABA from the whole blood of these HB prior to Imprime treatment abrogated Imprime binding and function. In subjects with low ABA levels (i.e. low binders or LB), binding and Imprime-induced function could be rescued with ABA supplementation, either in the form of sera from HB, enriched ABA or IVIG (Figs 4, 7 and 8). Parallels to this phenomenon can be drawn from ‘antibody-dependent enhancement (ADE)’ that occurs when non-neutralizing anti-viral antibodies facilitate entry into host cells, leading to enhanced infectivity in the cells. ADE has been demonstrated to enhance infectivity of viruses like Dengue and HIV through interactions of complement system and FcgR [23–25].

Consistent with immune complexes [26], Imprime-ABA binding primarily involved classical pathway of complement activation, while the alternative pathway had a partial role (Figs 2 and 3). Complement-dependent binding of Imprime was confirmed by the lack of binding in serum-depleted, heat-inactivated serum and CR1 blocking conditions. Significant abrogation of Imprime binding by anti-CR1 antibody is an important finding as it suggests that the deposition of iC3b opsonin on Imprime is critical to allow binding to CR3. As Imprime binding to CR3 is effectively blocked only by combination of the antibodies to the I-domain and the lectin domain of the CD11b chain of CR3, it is possible that Imprime binds to CR3 directly (lectin-domain ligand) as well as via the iC3b opsonin (I-domain ligand).

The Imprime-ABA immune complex is likely a mixture of IgG1- and IgG2-Imprime complexes, with IgG2-Imprime complex being predominant in most individuals (Fig 5). It is therefore not surprising that CD32A, the sole FcgR capable of binding multimeric IgG2 with higher affinity [17, 18], was demonstrated to play a critical role in binding of Imprime-ABA complex (Fig 5B–5D). It is also logical that the subjects separated out into HB and LB based on ABA concentration as a minimum epitope density is required for IgG2 to activate the classical pathway as well as to bind to FcgRIIA [15, 27].

Imprime-induced responses were consistent with the innate immune activation elicited by a pathogen (Fig 6). Imprime induced significant activation of complement proteins as well as phenotypic activation and chemokine production by innate immune cells. Although Imprime induced C5a production, no membrane attack complexes were detected on the cell surface suggesting that intravenous administration of Imprime should not result in uncontrolled complement activation and hemolysis of immune cells. Phenotypic activation included up-regulation of CD11b, and down-modulation of CD62L, CD88 (via interaction with C5a), and CXCR2, all of which are consistent with the activation state of neutrophils and monocytes that have encountered a pathogen and are primed to combat an infection [28–33]. Imprime significantly induced chemokines that have been shown to be critical for the adhesion of neutrophils and monocytes to the vascular endothelium during extravasation to the inflamed tissue [34, 35]. Imprime did not consistently induce the canonical pro-inflammatory cytokines IL-1β, TNF-α, and IL-6 that are triggered by other PAMPs [36, 37] Clinical experience has also confirmed that Imprime is well tolerated when administered systemically [38] and, thus, is a novel PAMP unique from other innate immunomodulators which cannot be safely administered systemically [39, 40].

Importantly, Imprime treatment also enhanced the functional response of these cells to a second stimulus (i.e., enhanced ROS production and ADCP by neutrophils and monocytes, respectively) upon interacting with tumor cells opsonized with tumor targeting antibodies. This finding is not surprising as enhanced oxidative burst and phagocytosis are among the several functional consequences of the activating signal generated from engagement of CR3 and activating FcgRs [41–43]. These anti-tumor cytotoxic mechanisms initiated by Imprime are important findings as the *in vivo* mouse tumor efficacy models had only delineated the critical role of CR3 and Gr1 expressing myeloid cells but not the precise effector mechanisms of tumor killing induced by combination of Imprime and tumor targeting antibodies [4, 5].

An important aspect of host recognition of a pathogen is the recognition of PAMPs by multiple pattern recognition receptors (PRRs) and their interplay in mediating a coordinated inflammatory response. Cooperation between CR3 and FcgR on neutrophils and monocytes has been previously demonstrated to play a role in pathogen recognition and immune activation [44–47]. It is possible that either both the receptors are simultaneously involved in binding of Imprime, or the engagement of FcgRIIA alone is first providing the inside-out signaling that brings about the conformational change to the high affinity form of CR3 receptor needed to bind Imprime [43, 48]. Although IL-8 production could be induced by Imprime-ABA in a complement-free system (washed WB), presumably via binding to FcgRIIA alone, robust induction of MCP-1 required intact complement (Fig 9). It is thus possible that either CR3 or FcgR could be driving specific functional responses, but on the whole, their cooperation is required for optimal binding and complete functionality of Imprime in WB (i.e. physiologically-relevant condition). Although Dectin-1 blocking antibody did not inhibit Imprime binding to neutrophils or monocytes [9], it is still formally possible that Dectin-1 is involved but not solely responsible for binidng. Furthermore, Dectin-1 signaling is known to be activated mainly by particulate and not soluble glucans like Imprime [49]. As such, it is possible that the tripartite Imprime-ABA-complement complex could signal through Dectin-1 cued in by CR3 and FcgR engagement, or vice versa. Another important player that could be critical in the cooperative recognition and signaling of Imprime is C5a. C5a is a potent inflammatory mediator that has been shown to induce a broad rage of effector functions via cross-talk with CR3 and FcgR, including oxidative burst and cytokine production [50–56]. Overall, further studies are warranted to understand the hierarchical or simultaneous signaling cross-talk between the receptors that are ultimately critical for Imprime’s anti-tumor activity.

In summary, the data in this report provide the first evidence that the novel β glucan immunotherapeutic, Imprime, is pharmacologically active as an immune complex formed by binding to naturally occurring ABA and subsequent opsonization by complement proteins. This three-part complex binds to and activates anti-cancer innate immune effector function through both CR3 and the FcgRIIa. The concentration of ABA in serum is a critical determinant of the ability of Imprime to bind and activate the immune cells from healthy subjects. Immune cells from human subjects with insufficient ABA do not respond to Imprime but can respond when supplemented with exogenous ABA. Collectively, these data suggest that responsiveness to Imprime-based immunotherapy is predicated on ABA levels in individual subjects. Accordingly, pre-treatment ABA levels may represent a plausible, minimally-invasive biomarker to enrich for patients most likely to benefit from Imprime-based immunotherapy.

## Supporting Information

S1 Fig Lectin and alternative complement pathways are not critical for Imprime binding to neutrophils and monocytes in whole blood. The role of the lectin and alternative complement activation pathways in Imprime binding to neutrophils and monocytes was evaluated by blocking MBL (A) and factor D (B), respectively. WB was incubated with anti-MBL (20 μg/mL) and anti-factor D (10 μg/mL) mAbs at 4°C for 30 mins prior to the incubation with 10 μg/mL Imprime or vehicle at 37°C for 30 mins. The MFI and percentage of BfD IV positive cells are indicated on the contour plots. Data shown are representative of 3 independent experiments.
(TIF)

S2 Fig Distribution curves of IgG and IgM ABA concentrations in HB and LB of Imprime. The figures showed the distribution of LB and HB for both neutrophils and monocytes across a range of IgG and IgM ABA concentrations (RAU/mL) of 143 healthy subjects.
(EPS)

S3 Fig Lower detection of Imprime binding in LB is not due to compromised complement activity or internalization. (A) The amount of CH50 in LB and HB was measured by ELISA. The graph represents amount of CH50 production induced by the activator provided by the kit and the value of CH50 is reported as unit equivalents per mL of 15 HB and 11 LB. (B) Binding of Imprime in WB of HB and LB was evaluated as described in S1 Fig at 4°C and 37°C.
(EPS)

S4 Fig Imprime does not induce C5b-9 formation on cell surface in whole blood. WB was incubated with Imprime (10 μg/mL), or vehicle for 30 mins at 37°C and surface-bound C5b-9 was then detected with the anti-C5b-9 mAb by flow cytometry. WGP (10 μg/mL) was used as a positive control. Neutrophils and monocytes were identified with anti-CD15 and anti-CD14 mAb, respectively. The MFI and percentage of BfD IV positive cells are indicated on the contour plots. Data shown are representative of 3 independent experiments.
(EPS)

S5 Fig Evaluation of Imprime binding to FcgRIIA alone or CR3 alone. (A) To evaluate binding to FcgRIIA alone, WB was washed 6 times in dPBS and then reconstituted with dPBS to the original volume (complement-free). The WB was incubated with blocking antibodies for CD16, CD32, CD64, or CR1 at 4°C for 30 mins prior to adding enriched ABA (200 RAU/mL) and Imprime. Imprime binding was assessed by flow cytometry as described above. (B) Imprime binding in a subject having higher IgM ABA concentration and IgG ABA concentration in the range of a low binder was assessed in the presence and absence of the FcgR and CR1 blocking antibodies by flow cytometry. The MFI and percentage of BfD IV positive cells are indicated on the contour plots. Data shown are representative of 3 independent experiments.
(EPS)

S1 Table Frequency table of ABA concentration and Imprime binding to neutrophils in 143 healthy subjects. (DOCX)

S2 Table Frequency table of ABA concentration and Imprime binding to monocytes in 143 healthy subjects. (DOCX)

S3 Table Cytokine analysis of Imprime-treated or TLR-7/8 agnoist-treated WB. (DOCX)
