# Supplementary figures and images for: Imprime PGG-Mediated Anti-Cancer Immune Activation Requires Immune Complex Formation
Source: PLoS One. 2016 Nov 3;11(11):e0165909. doi: 10.1371/journal.pone.0165909 (PMC5094785; doi:10.1371/journal.pone.0165909)

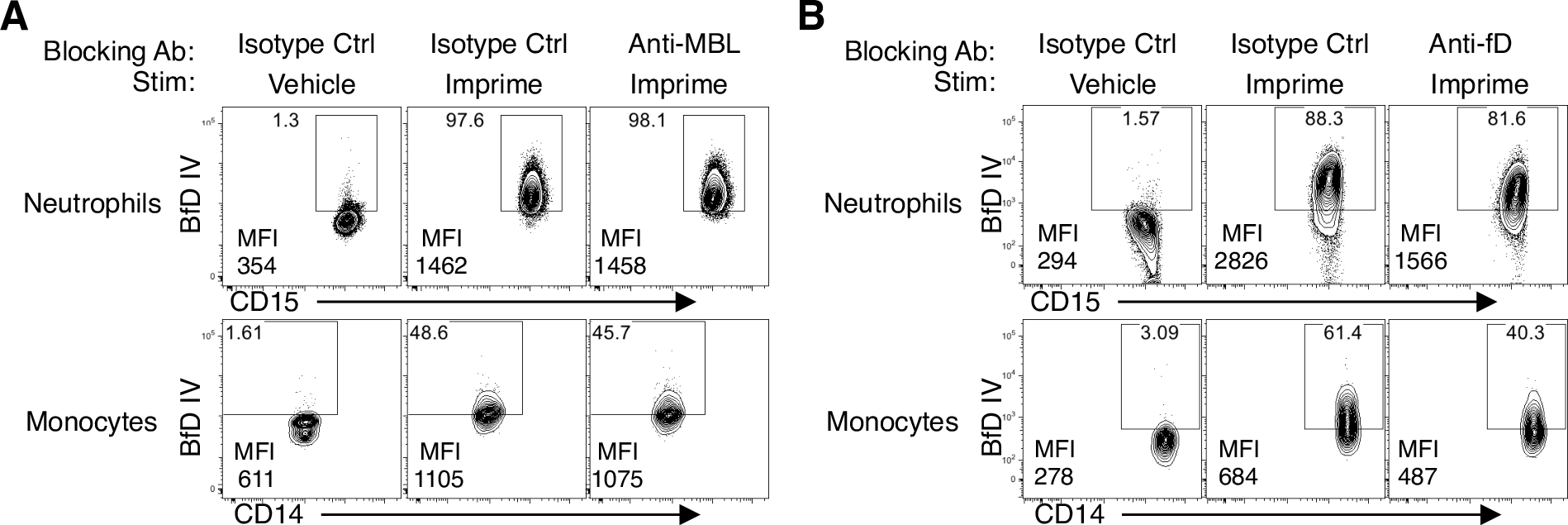

Supplement: S1 Fig — The role of the lectin and alternative complement activation pathways in Imprime binding to neutrophils and monocytes was evaluated by blocking MBL (A) and factor D (B), respectively. WB was incubated with anti-MBL (20 μg/mL) and anti-factor D (10 μg/mL) mAbs at 4°C for 30 mins prior to the incubation with 10 μg/mL Imprime or vehicle at 37°C for 30 mins. The MFI and percentage of BfD IV positive cells are indicated on the contour plots. Data shown are representative of 3 independent experiments. (TIF) [file pone.0165909.s001.tif]
