# Supplementary material for: Imprime PGG-Mediated Anti-Cancer Immune Activation Requires Immune Complex Formation
Source: PLoS One. 2016 Nov 3;11(11):e0165909. doi: 10.1371/journal.pone.0165909 (PMC5094785; doi:10.1371/journal.pone.0165909)
Supplement: S1 Table — (DOCX) [file pone.0165909.s006.docx]

**S1 Table. Frequency table of ABA concentration and Imprime binding to neutrophils in 143 healthy subjects.**

| **IgG Value**  **(RAU/mL)** | **Low Binders*** | | **High Binders** | | |  | **IgM Value**  **(RAU/mL)** | **Low Binders*** | | **High Binders** | | |
| --- | --- | --- | --- | --- | --- | --- | --- | --- | --- | --- | --- | --- |
|  | **Total Samples** | **Percent Samples** | **Total Samples** | **Percent Samples** | **Percent Binding Range** |  |  | **Total Samples** | **Percent Samples** | **Total Samples** | **Percent Samples** | **Percent Binding Range** |
| < 24.99 | 4 | 5.9 | 1 | 1.3 | 25.1 |  | < 24.99 | 4 | 5.9 | 2 | 2.7 | 29.7-58.4 |
| 25-74.99 | 16 | 23.5 | 1 | 1.3 | 10.3 |  | 25-74.99 | 22 | 32.4 | 13 | 17.3 | 7.7-74.7 |
| 75-124.99 | 15 | 22.1 | 3 | 4 | 7.3-44 |  | 75-124.99 | 22 | 32.4 | 12 | 16 | 5.2-80 |
| 125-174.99 | 14 | 20.6 | 8 | 10.7 | 6.3-84.8 |  | 125-174.99 | 7 | 10.3 | 6 | 8 | 6.3-34.2 |
| 175-224.99 | 9 | 13.2 | 4 | 5.3 | 29-70 |  | 175-224.99 | 5 | 7.4 | 14 | 18.7 | 8.2-97.9 |
| 225-274.99 | 5 | 7.4 | 5 | 6.7 | 8.5-74 |  | 225-274.99 | 4 | 5.9 | 3 | 4 | 24.4-74.7 |
| 275-324.99 | 1 | 1.5 | 4 | 5.3 | 6.3-70.2 |  | 275-324.99 | 3 | 4.4 | 9 | 12 | 10.3-82.2 |
| 325-374.99 | 3 | 4.4 | 2 | 2.7 | 65.6-79.3 |  | 325-374.99 | 0 | 0 | 3 | 4 | 7.3-92 |
| 375-424.99 | 0 | 0 | 2 | 2.7 | 24.1-34.2 |  | 375-424.99 | 0 | 0 | 5 | 6.7 | 6.3-84.8 |
| 425-474.99 | 0 | 0 | 6 | 8 | 14.4-55.6 |  | 425-474.99 | 1 | 1.5 | 0 | 0 | 0 |
| 475-524.99 | 0 | 0 | 2 | 2.7 | 26-92.5 |  | 475-524.99 | 0 | 0 | 1 | 1.3 | 25.1 |
| 525-574.99 | 0 | 0 | 6 | 8 | 6.5-97.9 |  | 525-574.99 | 0 | 0 | 1 | 1.3 | 39.5 |
| 575-624.99 | 1 | 1.5 | 8 | 10.7 | 19.9-93.9 |  | 575-624.99 | 0 | 0 | 1 | 1.3 | 49.4 |
| 625-674.99 | 0 | 0 | 3 | 4 | 8.2-74.7 |  | 625-674.99 | 0 | 0 | 1 | 1.3 | 93.9 |
| 675-724.99 | 0 | 0 | 4 | 5.3 | 46.1-92 |  | 675-724.99 | 0 | 0 | 1 | 1.3 | 90.3 |
| 725-774.99 | 0 | 0 | 0 | 0 | 0 |  | 725-774.99 | 0 | 0 | 0 | 0 | 0 |
| 775-824.99 | 0 | 0 | 0 | 0 | 0 |  | 775-824.99 | 0 | 0 | 0 | 0 | 0 |
| 825-874.99 | 0 | 0 | 5 | 6.7 | 5.2-90.3 |  | 825-874.99 | 0 | 0 | 0 | 0 | 0 |
| 875-924.99 | 0 | 0 | 2 | 2.7 | 55.2-60.1 |  | 875-924.99 | 0 | 0 | 0 | 0 | 0 |
| 925-974.99 | 0 | 0 | 1 | 1.3 | 72 |  | 925-974.99 | 0 | 0 | 0 | 0 | 0 |
| 975-1024.99 | 0 | 0 | 1 | 1.3 | 56.6 |  | 975-1024.99 | 0 | 0 | 0 | 0 | 0 |
| 1025-1074.99 | 0 | 0 | 2 | 2.7 | 53.4-85.6 |  | 1025-1074.99 | 0 | 0 | 0 | 0 | 0 |
| >1075 | 0 | 0 | 5 | 6.7 | 45.5-88.9 |  | >1075 | 0 | 0 | 3 | 4 | 34.8-56.7 |

*Percent binding range for Low Binders is consistently between 0 and 5%, and was not reported in the table.
