# Supplementary material for: Imprime PGG-Mediated Anti-Cancer Immune Activation Requires Immune Complex Formation
Source: PLoS One. 2016 Nov 3;11(11):e0165909. doi: 10.1371/journal.pone.0165909 (PMC5094785; doi:10.1371/journal.pone.0165909)
Supplement: S2 Table — (DOCX) [file pone.0165909.s007.docx]

**S2 Table. Frequency table of ABA concentration and Imprime binding to monocytes in 143 healthy subjects.**

| **IgG Value**  **(RAU/mL)** | **Low Binders*** | | **High Binders** | | |  | **IgM Value**  **(RAU/mL)** | **Low Binders*** | | **High Binders** | | |
| --- | --- | --- | --- | --- | --- | --- | --- | --- | --- | --- | --- | --- |
|  | **Total Samples** | **Percent Samples** | **Total Samples** | **Percent Samples** | **Percent Binding Range** |  |  | **Total Samples** | **Percent Samples** | **Total Samples** | **Percent Samples** | **Percent Binding Range** |
| < 24.99 | 4 | 7.7 | 1 | 1.1 | 27.2 |  | < 24.99 | 4 | 7.7 | 2 | 2.2 | 12.7-19 |
| 25-74.99 | 12 | 23.1 | 5 | 5.5 | 5.2-20 |  | 25-74.99 | 22 | 42.3 | 13 | 14.3 | 6.4-59 |
| 75-124.99 | 13 | 25 | 5 | 5.5 | 7.1-26.8 |  | 75-124.99 | 13 | 25 | 21 | 23.1 | 5.2-51.2 |
| 125-174.99 | 10 | 19.2 | 12 | 13.2 | 5.6-61.6 |  | 125-174.99 | 6 | 11.5 | 7 | 7.7 | 7.1-17.5 |
| 175-224.99 | 8 | 15.4 | 5 | 5.5 | 5.7-54.4 |  | 175-224.99 | 2 | 3.8 | 17 | 18.7 | 5.6-87.3 |
| 225-274.99 | 3 | 5.8 | 7 | 7.7 | 9.3-37.1 |  | 225-274.99 | 2 | 3.8 | 5 | 5.5 | 7.5-45 |
| 275-324.99 | 0 | 0 | 5 | 5.5 | 7.8-35.5 |  | 275-324.99 | 2 | 3.8 | 10 | 11 | 6.3-68.5 |
| 325-374.99 | 1 | 1.9 | 4 | 4.4 | 5.7-53 |  | 325-374.99 | 0 | 0 | 3 | 3.3 | 26.8-56.4 |
| 375-424.99 | 1 | 1.9 | 1 | 1.1 | 15.2 |  | 375-424.99 | 0 | 0 | 5 | 5.5 | 7.8-61.9 |
| 425-474.99 | 0 | 0 | 6 | 6.6 | 6.4-28.9 |  | 425-474.99 | 1 | 1.9 | 0 | 0 | 0 |
| 475-524.99 | 0 | 0 | 2 | 2.2 | 8.7-78 |  | 475-524.99 | 0 | 0 | 1 | 1.1 | 27.2 |
| 525-574.99 | 0 | 0 | 6 | 6.6 | 6.5-87.3 |  | 525-574.99 | 0 | 0 | 1 | 1.1 | 32.4 |
| 575-624.99 | 0 | 0 | 9 | 9.9 | 15.5-68.5 |  | 575-624.99 | 0 | 0 | 1 | 1.1 | 37.1 |
| 625-674.99 | 0 | 0 | 3 | 3.3 | 15.6-45 |  | 625-674.99 | 0 | 0 | 1 | 1.1 | 56.4 |
| 675-724.99 | 0 | 0 | 4 | 4.4 | 11.9-59 |  | 675-724.99 | 0 | 0 | 1 | 1.1 | 75.5 |
| 725-774.99 | 0 | 0 | 0 | 0 | 0 |  | 725-774.99 | 0 | 0 | 0 | 0 | 0 |
| 775-824.99 | 0 | 0 | 0 | 0 | 0 |  | 775-824.99 | 0 | 0 | 0 | 0 | 0 |
| 825-874.99 | 0 | 0 | 5 | 5.5 | 14.3-75.5 |  | 825-874.99 | 0 | 0 | 0 | 0 | 0 |
| 875-924.99 | 0 | 0 | 2 | 2.2 | 24.6-31.2 |  | 875-924.99 | 0 | 0 | 0 | 0 | 0 |
| 925-974.99 | 0 | 0 | 1 | 1.1 | 39.3 |  | 925-974.99 | 0 | 0 | 0 | 0 | 0 |
| 975-1024.99 | 0 | 0 | 1 | 1.1 | 21.8 |  | 975-1024.99 | 0 | 0 | 0 | 0 | 0 |
| 1025-1074.99 | 0 | 0 | 2 | 2.2 | 33-69.5 |  | 1025-1074.99 | 0 | 0 | 0 | 0 | 0 |
| >1075 | 0 | 0 | 5 | 5.5 | 21.5-81.5 |  | >1075 | 0 | 0 | 3 | 3.3 | 22.4-67 |

*Percent binding range for Low Binders is consistently between 0 and 5%, and was not reported in the table.
