# Supplementary material for: Imprime PGG-Mediated Anti-Cancer Immune Activation Requires Immune Complex Formation
Source: PLoS One. 2016 Nov 3;11(11):e0165909. doi: 10.1371/journal.pone.0165909 (PMC5094785; doi:10.1371/journal.pone.0165909)
Supplement: S3 Table — (DOCX) [file pone.0165909.s008.docx]

**S3 Table. Cytokine analysis of Imprime-treated or TLR-7/8 agnoist-treated WB.**

|  | **Cytokine (pg/mL)** | | |
| --- | --- | --- | --- |
|  | Vehicle | Imprime | TLR 7/8 Agonist |
| GM-CSF | 6.1 | 17.6 | 33.9 |
| IFN-γ | 1.8 | 3.6 | 629.5 |
| IL-10 | 1.8 | 2.2 | 3756.9 |
| IL-12 p70 | 2.3 | 2.5 | 183.9 |
| IL-13 | 3.2 | 1.7 | 2.1 |
| IL-1β | 1.2 | 10.8 | 12780.8 |
| IL-2 | 1.8 | 0.1 | 3.2 |
| IL-4 | 0.9 | 1.5 | 3.9 |
| IL-5 | 0.8 | 0.8 | 0.9 |
| IL-6 | 0.6 | 21.9 | 97926.8 |
| IL-7 | 5.0 | 10.3 | 31.5 |
| IL-8 | 832.6 | 3305.2 | 4618.0 |
| MCP-1 | 245.5 | 2849.7 | 11643.5 |
| TNF-α | 4.5 | 10.6 | 14973.2 |
